# Polarized Phase-Sensitive Fluorescence-Image Correlation Spectroscopy

**DOI:** 10.3390/biom16030433

**Published:** 2026-03-13

**Authors:** Andrew H. A. Clayton

**Affiliations:** Optical Sciences Centre, Department of Physics and Astronomy, School of Science, Computing and Emerging Technologies, Swinburne University of Technology, Hawthorn, VIC 3122, Australia; aclayton@swin.edu.au

**Keywords:** image correlation spectroscopy, fluorescence lifetime imaging microscopy, polarization

## Abstract

Molecular interactions underpin the functioning of the living cell. Molecules exist in distinct quaternary structural forms, associate with molecular partners in signaling cascades, form transient quinary interactions, localize in membrane domains, and cluster in membrane-less condensates. Measuring the concentration, size, and dynamics of these molecular assemblies remains an enduring biophysical challenge, particularly in cells, where heterogeneity is the rule rather than the exception. Orthogonal signals derived from fluorescence lifetime, fluorescence fluctuations, and fluorescence polarization provide valuable metrics for probing interactions and environments, concentration and size, and rotational dynamics, respectively. This paper combines fluorescence lifetime imaging microscopy with image correlation analysis and polarization to determine the concentrations, brightness, lifetime, and rotational correlation time of different fluorescent states. A two-population model is examined as a prototypical example of a heterogeneous system. The analysis is illustrated on a simple fluorescence model system, where cluster densities, relative brightnesses, lifetimes, and rotational correlation times are extracted.

## 1. Introduction

The living cell represents a fascinating entity for biophysical inquiry. At the molecular scale, proteins, nucleic acids, carbohydrates, and lipids form networks of transient interactions, polymers, membranes, organelles, membrane-less condensates, and machines capable of carrying out the instructions in the genetic blueprint. To function, molecules must move. Making sense of these thermally driven (non)equilibrium motions and interactions is a challenge, akin to the partially stochastic, partially random motions of people in a metropolis. At the same time, the invention of techniques such as fluorescence correlation spectroscopy [1] allows the experimentalist to extract quantitative information from the apparent noisy behavior of molecules in time observed through a tiny window (diffraction-limited excitation volume). If we instead take a wide-field snapshot from a population of these molecules in a single moment of time, we can still obtain information from the fluctuations in space. Analysis of fluctuations from an image is referred to as image correlation spectroscopy [2,3,4,5,6,7,8,9]. The power of images is the very large number of fluctuations that can be obtained in parallel.

Fluorescence itself is a phenomenon rich with information. The average time a molecule spends in the excited state or fluorescence (detected) lifetime, typically nanoseconds, is sensitive to the molecular environment. Lifetime measurements become especially valuable in methods such as Foerster Resonance Energy Transfer, where energy transfer from an initially excited donor molecule to a nearby (1–10 nm) acceptor molecule produces a characteristic proximity-dependent reduction in fluorescence lifetime [10,11,12,13,14,15,16]. Fluorescence polarization (or anisotropy) [17,18,19,20,21,22,23,24,25,26,27,28,29] is a dimension of fluorescence that is sensitive to rotational motion. Excitation with plane-polarized light produces an instantaneous anisotropic distribution of excited-state molecules with transition dipole moments aligned with the electric-field vector of the exciting light. Detection of the parallel and/or perpendicular components of the emission after polarized excitation reflects the ensuing change in orientation of the ensemble as the orientations become randomized through collisions with solvent molecules.

In this paper, we wish to use image correlation spectroscopy in a novel way. By combining image correlation spectroscopy with lifetime imaging microscopy and polarized excitation/detection, the goal is to determine the density, brightness, lifetime, and rotational correlation time of different species in an image. The key is the use of phase-sensitive detection in fluorescence lifetime imaging microscopy [10]. In wide-field frequency-domain FLIM, excitation is in the form of sinusoidally modulated light at radio frequencies (typically 40 MHz). The fluorescence is then detected with a 2D intensifier camera, which is gain-modulated at the same frequency as the fluorescence. By progressively shifting the phase of the detector with respect to the fluorescence, particles with a certain lifetime can be in-phase or out-of-phase with the detector. In this manner, particles with certain lifetimes can be optically suppressed or enhanced in the image. By using image correlation spectroscopy to measure the cluster density of particles as a function of detector phase, the densities of particles with different lifetimes can then be inferred [30]. When polarized excitation and orthogonal polarized detection are added to the lifetime measurement, fluorescence from slow-rotating species will be selectively diminished with respect to fast-rotating species, while species that rotate comparably with the excited-state lifetime will be further delayed in phase. By comparing the phase-dependent cluster densities in the unpolarized case with the polarized situation, information on rotational correlation times of different species should, in principle, be extractable.

The paper is organized as follows. In Section 3, we present the key equations underpinning the polarized phase-sensitive fluorescence image correlation spectroscopy method. In Section 4, we present simulations to show the sensitivity of the method. To provide a test bed for this novel ICS variant, we consider a two-population model as the simplest case for a heterogeneous system. Mimicking a receptor system in membrane domains, or a protein in different oligomeric states, we allow one population to be brighter than the other population. We simulate different combinations of lifetimes and correlation times. We then present measurements of phase-sensitive images with unpolarized excitation and polarized excitation of a model fluorescent system consisting of bright beads. In the context of a two-state model, we were able to recover the cluster densities, relative brightness, lifetimes, and rotational correlation times of the two states. In Section 5, we discuss the advantages and limitations of the new method.

## 2. Materials and Methods

Polarization and phase-dependent images were collected using a commercial frequency-domain FLIM system (LIFA, Lambert Instruments, Groningen, the Netherlands) mounted on a research-grade microscope (Model Ti, Nikon, Japan). Excitation was provided by a 474 nm LED modulated at 35 MHz, which was passed through a linear polarizer (Thor Labs, Newton, NJ, USA) and focused through a 4X objective lens (Nikon, Tokyo, Japan; NA = 0.1). Fluorescence was observed through a hyperspectral imaging system (His-400; Gooch & Housego, Orlando, FL, USA) set to 520 nm (20 nm width) and imaged using an intensifier-CCD camera. Our hyperspectral imaging system has a polarizer set to the horizontal (perpendicular), which serves as the analyzer in our microscope. The photocathode of the image intensifier was modulated at the same frequency as the excitation (35 MHz) but with a square-wave profile to increase instrument modulation depth. Twelve-phase images were recorded in pseudo-random order and over a full phase cycle under computer control using LI-FLIM v.1.2.12 software (Lambert Instruments, Groningen, The Netherlands). The exposure time per phase image was 500 ms. Instrumental correction factors were determined by using rhodamine 6G in distilled water (lifetime = 4.1 ns) as the reference, as previously described [30].

The image stack of 12 phase-dependent images was imported into FIJI for spatial autocorrelation analysis. Using the LI-FLIM software from Lambert Instruments, the reference stack and sample stack were imported as raw format images and then opened in the ImageJ v.2 (Fiji) open-source microscopy software program (NIH, Bethesda, MD, USA). Each image stack contained 12 phase-sensitive images. Regions of interest of size 256 × 256 pixels were selected and cropped. Average image intensities were calculated using the measure function in ImageJ. Autocorrelation analysis was performed using the correlation option in FD Math, and the resulting images were corrected by normalizing for the number of pixels and the square of the average image intensity and finally subtracting one. The inverse of the autocorrelation at zero lag was calculated as the cluster density or N in units of number per beam area.

Simulations of phase-sensitive fluorescence ICS and polarized phase-sensitive fluorescence ICS were in Microsoft Excel (Microsoft Corp., Redmond, WA, USA) using the equations outlined in Section 3 (Equations (3)–(9)) and Appendix A. Experimental N versus phase plots were fit to a theoretical model using least-squares minimization using the Solver function in Excel (Microsoft, USA). As models with increased complexity provide better fits to the experimental data, some justification for the increased number of parameters needs to be made. We used the Akaike Information Criterion (AIC), which allows models with different numbers of parameters to be compared and balances the need for a good fit to data with over-parameterization. The formula for the AIC value is given in Equation (1).

(1) AIC = 2k + nLN(LSQ)

where k is the number of parameters, n is the number of datapoints, LSQ is the sum of squared residuals, and LN is the natural logarithm.

## 3. Theory

The theory for phase-sensitive fluorescence image correlation spectroscopy was presented in a previous publication [30]. For the sake of completeness, we recap the most relevant formulae here and present the extension to polarized excitation/detection.

Recall that in our experimental fluorescence lifetime imaging microscopy set-up, fluorophores in an object are excited with intensity-modulated excitation (sinusoidal), and the fluorescence is detected by an image-intensifier-camera combination whose sensitivity is also (square wave) modulated at the same frequency as the excitation. In this homodyne mode of operation, fluorescence images are recorded at different phase settings of the detector over a full cycle of 0 to 2 pi. If we plot the average image intensity I as a function of detector phase ϑ, the intensity profile follows a cosine-like function; see Equation (2).

(2) I (ϑ) = I_0_ (1+ mcos(Φ − ϑ))

In the right-hand side of Equation (2), m is the modulation and Φ is the phase of the fluorescence signal, both corrected for the instrument response, and I_0_ is the average emission intensity. The modulation and phase values are related to the lifetime of the excited state of the fluorescence. If m = cos(Φ), then a unique lifetime may be determined from the phase or modulation values. If m< cos(Φ) then the excited-state decay process is more complex.

In the phase-sensitive fluorescence image correlation spectroscopy technique [30], image correlation spectroscopy analysis [4,5,6,7] is applied to fluorescence images recorded as a function of phase. In conventional ICS (4-5), the intensity fluctuation spatial autocorrelation function is computed from an image I(x,y) using a two-dimensional fast Fourier transform algorithm. The expression for the intensity fluctuation spatial autocorrelation function is

(3) $$g_{11}\left(\varepsilon ,\rho \right)=\frac{F^{-1}\left[F(I\left(x,y\right))*F^{*}(I\left(x,y\right))\right]}{{\left(\left|I\left(x,y\right)\right|\right)}^{2}}-1$$

where *F* represents the Fourier transform; *F*^−1^ is the inverse Fourier transform; F* is its complex conjugate; and the values *ε* and *ρ* are spatial lag variables.

The autocorrelation at zero-lag, g_11_(0,0), provides the measurement of the inverse mean number of particles per beam area and is obtained by fitting the spatial autocorrelation function to a two-dimensional Gaussian function.

(4) $$g_{11}\left(\varepsilon ,\rho \right)=g_{11}\left(0,0\right)exp\left[\frac{\varepsilon^{2}+\rho^{2}}{\omega^{2}}\right]+g_{\infty }$$

In Equation (4), g_∞_ is an offset to account for long-range spatial correlations, and ω is the full width at half maximum of the spatial autocorrelation function. For the remainder of the paper, we will refer to the autocorrelation at zero lag as simply g(0).

It is convenient to use the quantities cluster density or *N* and average brightness B as more intuitive measures of the particle distribution in an image. The cluster density can be readily obtained from the amplitude and width of the autocorrelation function, i.e.,

(5) $$N={\left(g\left(0\right)\pi \omega^{2}\right)}^{-1}$$

where *N* is the cluster density in units of number of particles per beam area.

In practice, noise and background can contribute to the cluster density determination, and corrections for these effects can be made as outlined by Petersen and Wiseman [4].

We now examine the microscopic factors behind the fluorescence image. Fluorescent entities are treated as particles with a brightness B (units: intensity/cluster) and dispersed with a cluster density N (units: clusters per beam area). If there is only one type of particle, then image correlation spectroscopy [4,5,6,7] analysis (spatial autocorrelation analysis) of the image will deliver the cluster density and brightness of that particle. For a heterogeneous system, image correlation spectroscopy analysis will deliver an average cluster density <N> as a weighted sum of the individual particle characteristics. To be more specific,

(6) <N> = (N_1_B_1_ + N_2_B_2_ + …)^2^/(N_1_(B_1_^2^) + N_2_(B_2_^2^) +…)

In Equation (6), N_1_ is the cluster density of population 1, B_1_ is the brightness of population 1, N_2_ is the cluster density of population 2, and B_2_ is the brightness of population 2, and so on.

In phase-sensitive fluorescence ICS [30], image correlation analysis [4,5,6,7] is applied to each image in the phase stack. We can rewrite Equation (6) as

(7) <N>(ϑ) = (N_1_B(ϑ)_1_ + N_2_B(ϑ)_2_ + …)^2^/(N_1_B(ϑ)_1_^2^ + N_2_B(ϑ)_2_^2^ + …)

where the brightness, B_i_ (ϑ), is modulated with the phase ϑ of the detector according to

(8) Bi (ϑ) = B_i_ (1 + mcos(Φ_i_ − ϑ))

As noted in our previous publication [30], if all particles have the same lifetime (phase), then the cluster density will be independent of the phase of the detector. However, if particles have different lifetimes, then the cluster density will be dependent on the phase of the detector. The output from the analysis of phase-sensitive ICS is the cluster density and brightness of particles with different lifetimes. The reader should refer to our previous paper [30] for examples of different types of cluster and lifetime distributions, including bimodal (two populations), Gaussian, and Lorentzian lifetime distributions.

We now wish to consider the situation of fluorescence lifetime imaging microscopy performed with polarized excitation and perpendicular polarized detection. Depopulation of the excited state will produce a phase shift and demodulation, as discussed above. Under conditions of polarized excitation and perpendicular polarized detection, rotational motion and/or transfer of electronic energy will induce an additional change in phase and hypo-modulate the emission [20]. We denote the resultant phase as Φ_perp_ and the modulation as m_perp_. The brightness will also decrease depending on the rotational correlation time (ϕ) and the lifetime (τ).

The form of the average cluster density of the perpendicular-polarized component of the emission (<N>(ϑ) _perp_) as a function of detector phase is analogous to the case of non-polarized excitation.

(9) <N> (ϑ) _perp_ = (N_1_B(ϑ)_1perp_ + N_2_B(ϑ)_2perp_ + …)^2^/(N_1_B(ϑ)_1perp_^2^ + N_2_B(ϑ)_2perp_^2^ + …)

where the brightness, B_i_ (ϑ)_perp_, is modulated with the phase ϑ of the detector according to

(10) B_i_ (ϑ) _perp_ = B_iperp_ (1 + m_perp_ cos(Φi_perp_ − ϑ))

For a fluorescent particle with anisotropy, r, the brightness of the particle is reduced with crossed polarization. The ratio of B_perp_ to B is given by Equation (11).

(11) B_perp_/B = (1/3) (1 − r)

The steady-state anisotropy is related in turn to the lifetime and correlation time by the Perrin–Jablonski equation,

(12) r = r_0_/(1 + (τ/ϕ))

where r_0_ is the fundamental anisotropy (in the absence of rotation). The perpendicular-polarized modulation and the perpendicular-polarized phase are complex functions of lifetime, correlation time, fundamental anisotropy, and optical modulation frequency and can be calculated from formulae published elsewhere [20,25,28,29]. For the interested reader, we present the formulae in Appendix A. In the next section, we investigate how different particle distributions influence the N and N_perp_ versus phase plots.

## 4. Results

In this section, we investigate different particle distributions, specifically examining different links between lifetime and correlation time. We begin with a simple system containing two populations of particles. One population with density N_1_ = 0.40 clusters/beam area and B = 3 (population 1), and a second population of particles with N_2_ = 0.39 clusters/beam area and B = 1 (population 2). The results allow some qualitative observations to be made.

(i) Single lifetime and single correlation time

**Figure 1:**
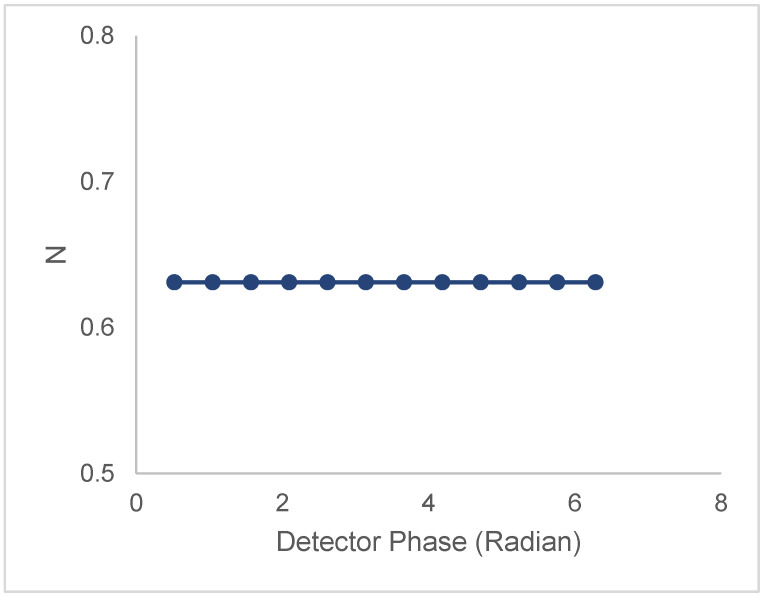
Polarized phase-sensitive fluorescence ICS for two-population model with identical lifetimes and identical correlation times (population 1 (N_1_ = 0.40 clusters/beam area; B = 3, τ = 20.2 ns, ϕ = ϕ_1_ = 5 ns); population 2 (N_2_ = 0.39 clusters/beam area, B = 1, τ = 20.2 ns, ϕ = ϕ_2_ = 5 ns)). The line with filled symbols represents the N_perp_ versus phase for the condition specified above. Simulations were with an optical modulation frequency of 35 MHz and a zero-time fundamental anisotropy r_0_ = 0.4.

Figure 1 depicts a simulation with all particles having a lifetime of 3.6 ns and a rotational correlation time of 5 ns. Despite the differences in particle densities and particle brightnesses between populations 1 and 2, the phase-dependent cluster densities are independent of phase and polarization. This is because all particles have an identical phase. The observed N is not equal to the sum of N_1_ and N_2_ because population 1 with N_1_ is three times brighter than population 2 with N_2_. The conclusion from this simulation is that to have an N that depends on phase, you must have some heterogeneity in phase (originating from heterogeneity in lifetime and/or correlation time). As an aside, for a single lifetime, single correlation time system, use of the modulation and phase (Equation (1)) from the average image intensity phase stack can be combined with the analogous quantities under polarized excitation/perpendicular polarized detection to extract the lifetime and correlation time using AB, polar, or phasor plot [28,29].

(ii) Single lifetime and two correlation times

**Figure 2:**
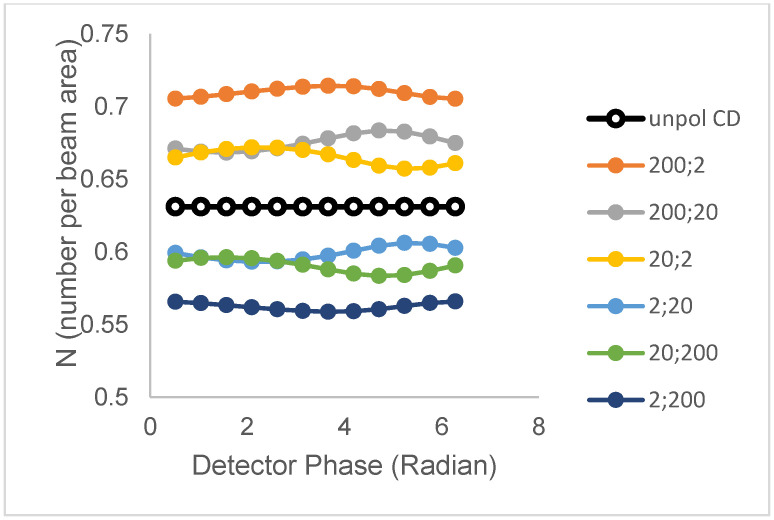
Polarized phase-sensitive fluorescence ICS for two-population models with identical lifetimes but different rotational correlation times (population 1 (N_1_ = 0.40 clusters/beam area; B = 3, τ = 20.2 ns, ϕ = ϕ_1_); population 2 (N_2_ = 0.39 clusters/beam area, B = 1, τ = 20.2 ns, ϕ = ϕ_2_)). Lines with filled symbols from top to bottom correspond to correlation time pairs (ϕ_1_; ϕ_2_) = (200; 2), (200; 20), (20; 2), (2; 20), (20; 200), and (2; 200). The line with unfilled symbols corresponds to unpolarized excitation. Simulations were with an optical modulation frequency of 35 MHz and a zero-time fundamental anisotropy r_0_ = 0.4.

We now examine the cases of population 1 (N_1_ = 0.40 clusters/beam area; B = 3, τ = 20.2 ns) and population 2 (N_2_ = 0.39 clusters/beam area, B = 1, τ = 20.2 ns) having distinct single correlation times, ϕ_1_ (for population 1) and ϕ_2_ (for population 2) but maintaining identical lifetimes. Figure 2 depicts simulations for different combinations of correlation times (filled symbols), together with the simulation for the unpolarized excitation (unfilled symbols). Rotational motion in both populations is seen to influence the N_perp_ versus phase plots for the perpendicular polarized emission. While the unpolarized excitation plot is linear with a gradient of zero (horizontal), the polarized plots show a small level of curvature. However, the curvature is low, with a coefficient of variation of less than 1%. The main effect of rotational motion is to produce an offset along the apparent N axis, relative to the unpolarized case. Conditions that make population 1 polarized and population 2 relatively depolarized give rise to an enhancement in the N_perp_ value (relative to the unpolarized case); whereas, conditions that make population 2 polarized and population 1 depolarized give rise to a reduction in Nperp value (relative to the unpolarized case). The curves show that a distinction between different correlation time models involving pairs of correlation times can be made, at least when the correlation times differ by an order of magnitude.

(iii) Two lifetimes and one correlation time

**Figure 3:**
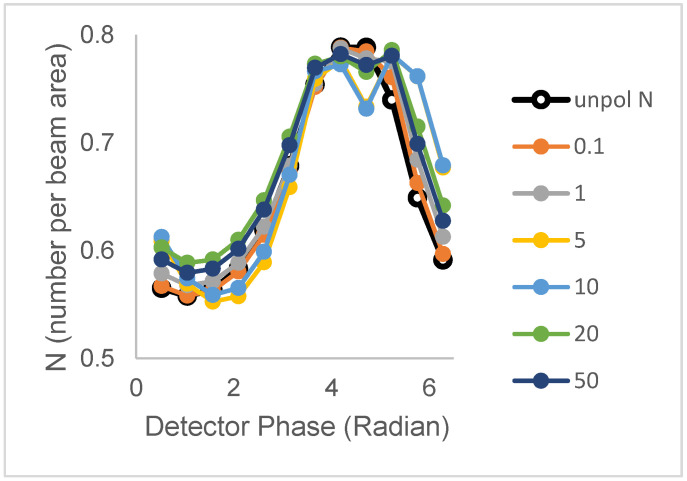
Polarized phase-sensitive fluorescence ICS for two-population models with distinct lifetimes but shared single rotational correlation times (population 1 (N_1_ = 0.40 clusters/beam area; B = 3, τ = 3.6 ns, ϕ = ϕ_1_); population 2 (N_2_ = 0.39 clusters/beam area, B = 1, τ = 20.2 ns, ϕ = ϕ_1_)). Lines with filled symbols from top to bottom at 6.28 radians correspond to correlation times (ϕ) = (10, 20, 50, 1, 0.1) in nanoseconds. The line with unfilled symbols corresponds to unpolarized excitation. Simulations were with an optical modulation frequency of 35 MHz and a zero-time fundamental anisotropy r_0_ = 0.4.

We now examine the cases of population 1 (N_1_ = 0.40 clusters/beam area; B = 3, τ_1_ = 3.6 ns) and population 2 (N_2_ = 0.39 clusters/beam area, B = 1, τ_2_ = 20.2 ns), having distinct single lifetimes but maintaining shared, single rotational correlation times. Figure 3 depicts the N_perp_ versus phase for different rotational correlation times. For the unpolarized case, the N versus phase plot has a distinctive peak, owing to the difference in lifetimes of the two populations and the larger brightness of the short-lifetime population. Rotational motion is seen to cause either broadening or peak splitting in the N_perp_ versus phase plots. Broadening occurs for short (0.1 and 1 ns) and very long correlation times (20 ns and 50 ns), while peak spitting occurs with a rotational correlation time of 5 ns and 10 ns.

(iv) Two lifetimes and two correlation times (un-associated)

**Figure 4:**
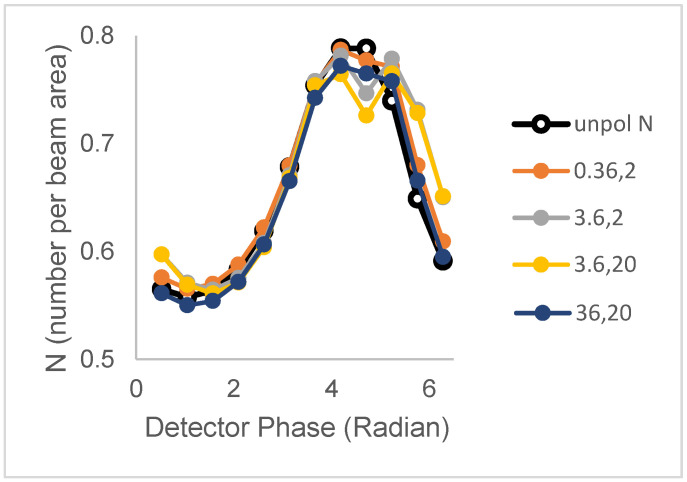
Polarized phase-sensitive fluorescence ICS for a 2-population model with distinct lifetimes but shared two-component rotational correlation times ((ϕ_1_, ϕ_2_)) (population 1 (N_1_ = 0.40 clusters/beam area; B = 3, τ_1_ = 3.6 ns); population 2 (N_2_ = 0.39 clusters/beam area, B = 1, τ_2_ = 20.2 ns). Lines with filled symbols from top to bottom at 4.7 radians correspond to correlation time pairs (ϕ_1_, ϕ_2_) in ns) = (0.36, 2), (36, 20), (3.6, 2), and (3.6, 20). The line with unfilled symbols corresponds to unpolarized excitation. Simulations were with an optical modulation frequency of 35 MHz and a zero-time fundamental anisotropy r_0_ = 0.4.

We now examine the cases of population 1 (N_1_ = 0.40 clusters/beam area; B = 3, τ_1_ = 3.6 ns) and population 2 (N_2_ = 0.39 clusters/beam area, B = 1, τ_2_ = 20.2 ns) having distinct single lifetimes but now sharing the same rotational correlation times (with a 50% contribution of ϕ_1_ and a 50% contribution of ϕ_2_). Figure 4 depicts the N versus phase for different value sets of ϕ_1_ and ϕ_2_: (0.36 ns, 2 ns), (3.6 ns, 2 ns), (3.6 ns, 20 ns), (0.36 ns, 20 ns), (36 ns, 2 ns), and (36 ns, 20 ns).

For the unpolarized case, the N versus phase plot has a distinctive peak, owing to the difference in lifetimes of the two populations and the larger brightness of the short-lifetime population. As for the single correlation time case (above), rotational motion is seen to cause either broadening or peak splitting in the N_perp_ versus phase plots.

(v) Two lifetimes and two correlation times (associated)

We now examine the cases of population 1 (N_1_ = 0.40 clusters/beam area; B = 3, τ_1_ = 3.6 ns) with distinct correlation time ϕ_1_ and population 2 (N_2_ = 0.39 clusters/beam area, B = 1, τ_2_ = 20.2 ns) with correlation time ϕ_2_. That is, an associative model.

**Figure 5:**
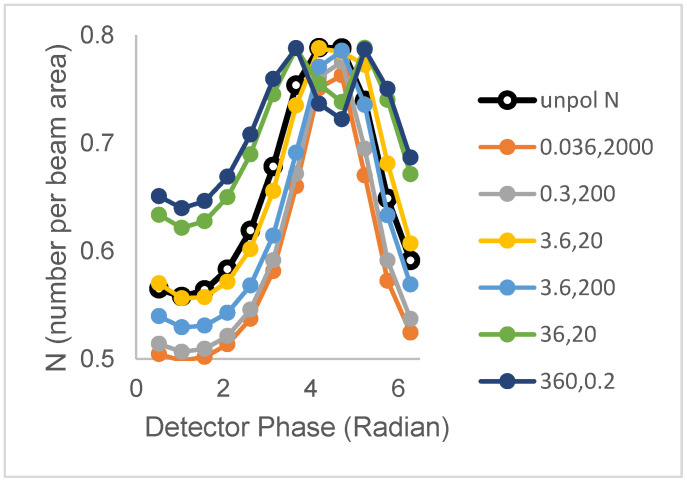
Polarized phase-sensitive ICS for two-population models with distinct lifetimes and associated distinct rotational correlation times (population 1 (N_1_ = 0.40; B = 3, τ_1_ = 3.6 ns); population 2 (N_2_ = 0.39, B = 1, τ_2_ = 20.2 ns). Lines with filled symbols from top to bottom at 6.28 radians correspond to lifetime-associated correlation times (τ_1_→ϕ_1_, τ_2_→ϕ_2_) = (360, 0.2), (36, 2), (3.6, 20), (3.6, 200), (0.36, 200), (0.036, 2000). The line with unfilled symbols corresponds to unpolarized excitation. Simulations were with an optical modulation frequency of 35 MHz and a zero-time fundamental anisotropy r_0_ = 0.4.

Figure 5 depicts N versus phase plots for different associative models. In the associative model, peak narrowing, peak broadening, and peak splitting are observed. However, there are also changes in the amplitude. Peak narrowing is accompanied by a decrease in peak amplitude, and peak splitting is accompanied by an increase in amplitudes of the two peaks. By way of orientation, the plot with the largest splitting and highest amplitudes corresponds to ϕ_1_ = 100τ_1_ = 360 ns and ϕ_2_ = 0.01τ_2_ = 0.2 ns; whereas, the plot with the single peak at the lowest amplitude corresponds to ϕ_1_ = 0.01τ_1_ = 0.036 ns and ϕ_2_ = 100τ_2_ = 2000 ns.

From the simulations, we can summarize the qualitative features of polarized phase-sensitive ICS simulations in Table 1 below. In general, homogeneous lifetimes and correlation times are predicted to produce N (N_perp_) versus phase plots that are independent of phase, lifetime, and correlation time. Single lifetimes but heterogeneous correlation times produce N_perp_ plots that depend on the rotational correlation times. Heterogeneous lifetimes produce dips (previous work [30]) or peaks in N with phase. Depending on how the rotational correlation times are coupled to the lifetimes (associative or non-associative), rotational motion can cause broadening, peak splitting, and amplitude changes in the N versus phase plots. These simulations provide some insights into the sorts of changes that might be anticipated where populations differ in brightness, lifetime, and rotational correlation times, but of course are not intended to be exhaustive.

(vi) Application to experimental sample

**Figure 6:**
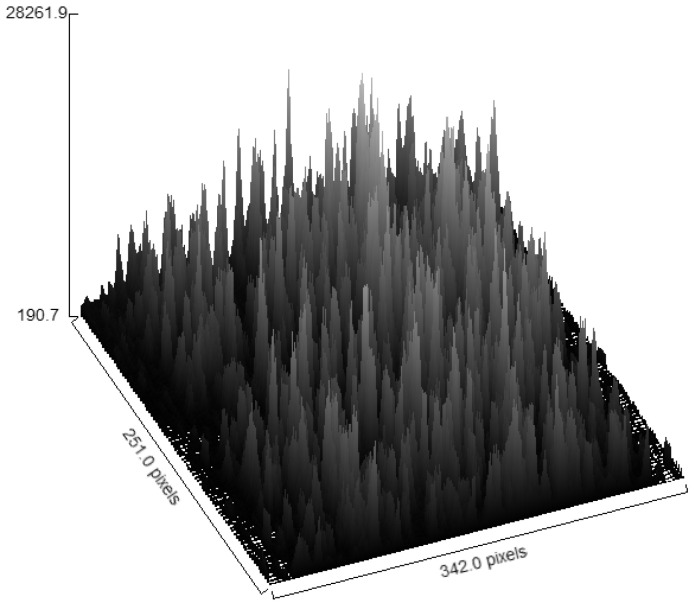
3D surface plot of a collection of fluorescent beads. Note the significant fluorescent fluctuations due to the fluorescent particles in the image. Images were acquired with 470 nm excitation and emission collected through a hyperspectral imaging device centered near 510 nm (width: 20 nm). Note: the 3D surface plot is for qualitative visualization purposes only.

To put into practice the ideas presented above, we require a sample that contains fluorescence fluctuations across an image. A test sample was created containing commercial fluorescent beads that were dropped onto a microscope slide and left to dry. Figure 6 depicts a surface plot (generated in FIJI) of a fluorescence image of the bead sample collected with a wide-field fluorescence microscope (LED excitation of 470 nm; emission filter centered at 510 nm) showing the anticipated punctate fluorescence from the beads.

As part of the standard operation of our commercial wide-field frequency-domain microscope (LIFA, Lambert Instruments, The Netherlands), phase-sensitive images were acquired at twelve different phases of the detector (optical modulation frequency, 35 MHz, and 12 phase steps over a 2 pi radians), under computer control. One series was collected without an excitation polarizer (unpolarized excitation), and the other series was collected with an excitation polarizer included in the excitation path of the microscope. Phase-dependent images were analyzed as described in Materials and Methods, and the output was displayed as N versus phase plots.

**Figure 7:**
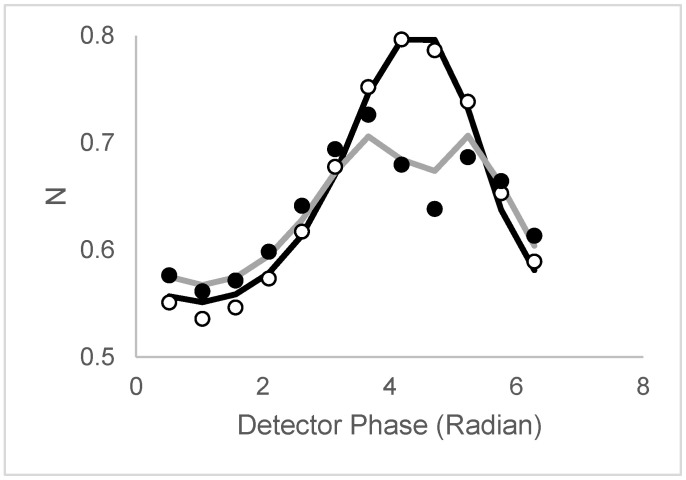
Polarized phase-sensitive fluorescence ICS from a fluorescence image of beads. Unfilled circles represent N versus phase with unpolarized excitation, and filled circles represent N_perp_ versus detector phase with polarized excitation. Solid black line represents the best fit to the unpolarized excitation data (parameters: (population 1 (N_1_ = 0.38; B_1_ = 3, τ_1_ = 3.9 ns); population 2 (N_2_ = 0.42, B_2_ = 1, τ_2_ = 19.4 ns). Gray solid line represents the best fit to the polarized-excitation data (population 1 (N_1_ = 0.38; B_1_ = 3, τ_1_ = 3.9 ns, ϕ_1_ = 1000 ns)); population 2 (N_2_ = 0.42, B_2_ = 1, τ_2_ = 19.4 ns, ϕ_1_ = 0.001 ns)).

Figure 7 depicts the N versus phase data (empty symbols) and the N_perp_ versus phase data (filled symbols) extracted from the bead images.

The N versus phase has a clear dependence on detector phase, with a trough near 1 radian of 0.53 clusters/beam area and a single peak near 4 radians close to 0.8 clusters/beam area. The N versus phase data was described with various models of increasing complexity. A summary of the models tested is collected in Table 2. To compare the different models, we used the Akaike Information Criterion (AIC), which balances the need for a good fit to the data with over-parameterization.

A model with a mono-disperse population yielded an average cluster density N = 0.65 clusters per beam area and a fluorescence lifetime of 5.2 ns. As expected, this model did not reproduce the phase-dependent peak shown in Figure 7 (in the model, N was independent of phase). The next model allowed for two populations with different densities and different lifetimes but identical brightness (B_1_ = B_2_ = 1). This model produced a fit that assigned both lifetimes to identical values and became equivalent to the one-population model. Because this model had two additional parameters, the AIC value was not improved relative to the simpler one-population model. The third model maintained the assumption of two populations with different lifetimes and cluster densities but relaxed the requirement of equal brightness in the two populations. The third model gave an improved AIC value of −113 (5 variable parameters) versus the first model, AIC = −45 (2 variable parameters), and the second model with AIC = −41 (4 variable parameters). The solid line in Figure 7 reveals the quality of fit to a two-population model for phase-sensitive fluorescence ICS (N versus phase data), with parameters (population 1 (N_1_ = 0.38; B_1_ = 3, τ = 3.9 ns); population 2 (N_2_ = 0.42, B_2_ = 1, τ = 19.4 ns)). Clearly, this model can reproduce the peak observed, while simpler models were unable to do so.

Now turning to the experimental polarized phase-sensitive fluorescence ICS data in Figure 7. The N_perp_ versus phase plot differs noticeably from the N versus phase plot, especially with split peaks and reduced peak amplitudes (Figure 7, filled circles). To fit the polarized phase-dependent ICS data, lifetimes and brightness values were fixed to the values obtained from the unpolarized excitation dataset, while N values were allowed to be rescaled by a constant factor. Different correlation time models were tested, including common single correlation time, common two correlation times, and an associative model. Table 3 provides a summary of the models tested, together with the AIC parameters.

The simplest anisotropy decay model is characterized by a single rotational correlation time. The first model assumed that both populations 1 and 2 rotated with the same rotational correlation time. The fit to this model yielded a common single correlation time of 25.9 ns and an AIC value of −66. The second most complex anisotropy decay model contains two rotational correlation times, ϕ_1_ and ϕ_2_. If we assume that population 1 rotates with correlation time ϕ_1_ and population 2 rotates with correlation time ϕ_2,_ then we have what is essentially known as an associative anisotropy decay model. Interestingly, this model, with the addition of one extra adjustable parameter, improved the AIC considerably to −90. The solid gray line in Figure 7 reveals the fit to an associative model. In this model, the 3.9 ns bright clusters are rotationally immobile (ϕ_1_ = 10,000 ns), while the dimmer 20 ns clusters are completely and rapidly depolarized (ϕ_2_ = 0.001 ns). Attempts to improve the model fits by including more parameters did not result in significant improvements. For example, we tested a model where each population had two rotational correlation times but different population-dependent proportions. The fits were not improved, and the AIC values were worse than the associative two-correlation-time model discussed above.

A self-consistency test of the model can be made by comparing the measured cluster density in a conventional (unmodulated) fluorescence image of the beads with the calculated cluster density derived from the extracted brightness and cluster densities of the populations. The measured cluster density of an unmodulated image (i.e., an image from summing the phase-dependent images over a full cycle) was 0.629 clusters/beam area. This can be compared with the value of 0.623 clusters/beam area derived from parameters (N_1_ = 0.38; B_1_ = 3.2; N_2_ = 0.43, B_2_ = 1) and Equation (3). Note that the difference between experiment and calculation is 1%, which is within the standard deviation of our measurement. A cluster density measurement under polarized excitation/perpendicular detection yielded a value of 0.635 clusters/beam area. Assuming population 1 is polarized (r = 0.4; N_1_ = 0.33; B_1_ = 1.9) and population 2 is depolarized (r = 0; N_2_ = 0.37, B_2_ = 1), we calculate a cluster density of 0.641 clusters/beam area, again within 1% of the experimental value, using Equations 3 and 8. Significantly, if we assume population 1 was depolarized (r = 0; N_1_ = 0.33; B_1_ = 3.2) and population 2 polarized (r = 0.4; N_2_ = 0.37, B_2_ = 0.6), we calculate a cluster density of 0.469 clusters/beam area. If both populations were equally polarized (N_1_ = 0.33; B_1_ = 3.2; N_2_ = 0.43, B_2_ = 1), we calculate a cluster density of 0.548 clusters/beam area. Thus, qualitatively, at least, a model with a polarized bright population and a depolarized dim population is consistent with the apparent cluster densities acquired from a conventional (unmodulated) fluorescence image of the beads.

A comparison can also be made between the extracted lifetimes from the phase-sensitive fluorescence ICS analysis and a two-component lifetime model from the FLIM image of the bead sample [12,13,14]. Using the polar plot feature in the LIFA software, and assuming only 2 lifetime states in the system, the extracted lifetimes were in the ranges 3.96–3.98 ns and 16.2–21.8 ns (range represents different intensity threshold settings). These estimates agree to within 20% of the lifetimes 3.9 ns and 19.4 ns determined with the phase-sensitive ICS approach. We note that the longer lifetime estimate is somewhat dependent on the choice of intensity threshold when analyzing the FLIM data-this appears to be a trade-off between improving signal to noise (higher threshold) and including the dimmer states (lower threshold). In the phase-sensitive fluorescence ICS approach, no manual thresholds were applied, nor are they normally required in ICS.

It is instructive to compare the results using our frequency-domain approach with the more conventional and intuitive time-resolved anisotropy decay. For this purpose, we simulated the anisotropy decay curve of the associative system. To do this, we use the more general formula [31] for the anisotropy decay of a two-population system, as

(13) r(t) = (I_1_(t)r_1_(t) + I_2_(t)r_2_(t))/(I_1_(t) + I_2_(t))

Equation (13) can be rewritten in terms of the parameters in this study; see Equation (13).

r(t) = Numerator(t)/Denominator(t)

Numerator(t) = [N_1_B_1_exp(−t/τ_1_)r0_1_exp(−t/ϕ_1_) + N_2_B_2_ exp(−t/τ_2_)r0_2_exp(−t/ϕ_2_)]

(14) Denominator(t) = N_1_B_1_exp(−t/τ_1_) + N_2_B_2_exp(−t/τ_2_)

**Figure 8:**
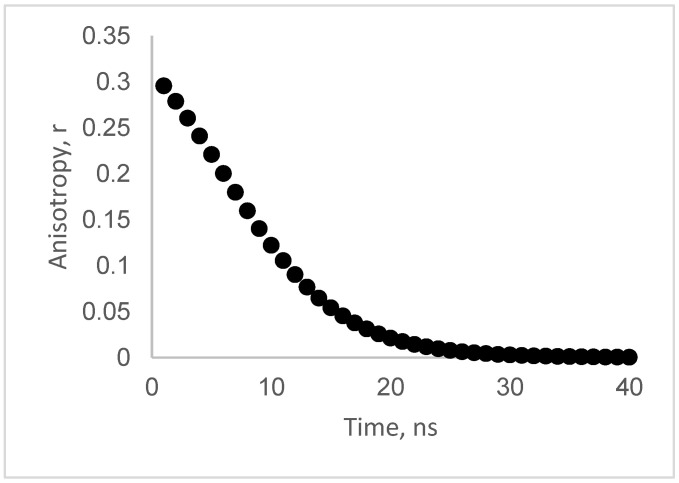
Anisotropy decay simulated from the two-population associative model assuming spatial averaging. Note that the associative model predicts an apparent anisotropy decay on the scale of nanoseconds, even though both populations are immobile. The plot was generated using Equation (11) with parameters (N_1_ = 0.38; B_1_ = 3, τ = 3.9 ns, r_01_ = 0.4, ϕ_1_ = 10,000 ns); population 2 (N_2_ = 0.42, B_2_ = 1, τ = 19.4 ns, r_02_ = 0, ϕ_2_ = 10,000 ns).

Figure 8 depicts a simulation using Equation (13) using the parameters extracted from the polarized phase-sensitive fluorescence ICS. The anisotropy decays from an initial value of 0.3 to nearly zero over a time range of tens of nanoseconds. A rough estimate of the average, apparent correlation time from this plot is about 10 ns.

## 5. Discussion

The goal of quantitative image analysis, seen from a molecular spectroscopy point of view, is to determine the molecular concentrations (or densities) of different states (defined by excited-state decay rates and diffusion parameters) and, in systems undergoing molecule-molecule interactions, the association states (brightness). Imaging or time-resolved spectroscopy alone cannot address all these requirements. Image population distribution is measured by pixel count, while time-resolved spectroscopy without spatial resolution provides data as photon counts per state.

The philosophy presented in this paper is to view an image of fluorescent particles as a spatial record of fluctuations. In this approach, the concentration and brightness information are determined from a spatial autocorrelation analysis of the images. This is the image correlation spectroscopy approach originally conceived and applied by Peterson and Wiseman [2,3,4,5,6,7,8,9]. By adding polarized illumination, modulated excitation, and modulated and polarized detection, phase-sensitive fluorescence images can be created that encode information on the excited-state and rotational rates. Image correlation analysis on the polarization and phase-sensitive fluorescence images can then be fit to a model for the underlying states in terms of concentration, brightness, lifetime, and correlation time.

The output from polarized phase-sensitive fluorescence image correlation spectroscopy is the (apparent) cluster density as a function of (detector) phase. A 3D dataset (I(x,y), phase) is collapsed onto a 2D plot (N, phase). The information gleaned from the plot is directly related to the heterogeneity in population and/or polarization decay rates (lifetimes and correlation times). An N independent of phase implies homogeneity of lifetimes in the image, while an N versus phase plot that shows a distinct dip [30] or peak (this work) implies a heterogeneous lifetime system. Heterogeneity in rotational motion can be manifested in changes to the shape (broadening and peak splitting) and/or amplitude of the N_perp_ versus phase plots relative to the unpolarized conditions.

When tested on a model fluorescence system, fluorescent beads dispersed on a microscope slide, and clear evidence for lifetime heterogeneity was obtained. In the context of an admittedly simple two-population model, we were able to extract the cluster densities and relative brightness of the two lifetime states. Extension to polarized excitation/perpendicular polarized detection enabled distinction between different correlation time models. Non-associative models (rotational motions common to both populations) could be clearly excluded in the analysis. An associative model with a highly polarized emission linked to one state and a highly depolarized emission linked to the other state was seen to be the best of the tested models.

It is important to discuss the advantages and limitations of the method presented here. The advantages and limitations of ICS are well understood from the papers from the Wiseman laboratory. Averaging over many fluctuations over a large area results in a robust estimate for particle densities [7]. Our replicate measurements on the fluorescent bead samples yielded standard deviations of 0.005 for cluster densities of the order of 0.5 clusters/beam area or about 1% coefficient of variation (120 separate measurements). Because fluorescent beads are ideal samples, the standard deviation reported here is likely to be a lower limit and thus an overestimate of the precision. The key assumption in the method presented here is that the actual density of particles is independent of the detector phase and that the brightness of different dynamic states is modulated with the detector phase, depending on the lifetime and/or correlation time characteristics. In principle, particle motion, photobleaching, or photo-induced lifetime or rotation would invalidate the assumptions presented above. Particle motion due to diffusion will produce fluctuations in the particle occupancy numbers in the image. However, depending on the particle density and the image size, these fluctuations can be estimated and considered in the analysis. Photobleaching will reduce intensities and cluster densities [32]. This can be estimated using an unmodulated sample and corrected in the analysis [32]. Photoconversion leading to changes in lifetime can be determined using standard lifetime measurements. We note that the assumptions in our method are less stringent than the assumptions made in camera-based fluorescence lifetime imaging microscopy. In FLIM, the assumption is that the number of molecules per pixel does not change during image acquisition, whereas in our method, the assumption is that the total number of particles per imaging area remains constant during image acquisition.

Regarding the polarized measurements, the approach to compute the cluster density under polarized excitation/perpendicular polarized detection is, to the best of our knowledge, novel. An advantage of using ICS is that it eliminates the need to measure a G-factor. This is primarily because the cluster density is computed as a relative squared fluctuation and is independent of the absolute signal intensity. Likewise, the phase and modulation are also parameters that are independent of the signal. Our method does not require calculations of anisotropy, phase difference, or modulation ratios that are conventionally used in frequency-domain anisotropy decay measurements [24,25,26,27]. The anisotropy, the difference in phase between parallel and perpendicular components of the emission, and the AC ratio between parallel and perpendicular components have been used as parameters to construct rotational correlation time images [24]. In contrast, our method seeks to provide a state representation of species in an image based on only a few components (cluster density, brightness, lifetime, and correlation time). Once the state representation is established, the spatial distribution of these different states can be mapped into an image. For example, in the context of associative lifetime–correlation time models, the phasor plot (or polar or AB plot) can be used to create images based on the fractional fluorescence of different lifetime states. Because of the lifetime–correlation time association, these images could then be used to create rotational correlation time images.

We envision a range of biological applications in which the polarized-phase-sensitive fluorescence ICS could be employed. Dyes [33,34,35,36], which can display environment-sensitive fluorescence, often have different lifetimes depending on the environment. Moreover, dyes can partition into domains, membranes, organelles [33], or even cells depending on biological/biophysical states [33,34,35,36]. This partition can create local structures with widely varying brightness and density. The partitioning or binding can also lead to a decrease in rotational diffusion or an increase in depolarization if dyes are packed together and undergo energy migration. Biological macromolecules often exhibit a complex “lifestyle” in the cellular environment. Interactions with other molecules can lead to altered lifetimes and correlation times, and self-association or localization in domains can lead to increases in brightness and/or density. FRET is another potential application [37,38,39,40]. FRET results in a decrease in the lifetime and quantum yield of the excited donor when it transfers non-radiatively to the acceptor. If FRET results from the interaction of one molecule with another, an increase in rotational correlation time of the donor should be observed due to the slower tumbling of the complex. Thus, molecule-molecule interactions can be probed with greater detail, given brightness, lifetime, and rotational correlation time information.

## 6. Conclusions

A novel extension to ICS, called polarized-phase-sensitive fluorescence ICS, was presented. Use of polarized excitation and perpendicular detection, as well as phase-sensitive detection, allows fluorescence from species with different lifetimes or rotational correlation times to be enhanced or diminished. Analysis of the fluctuations in space of images collected as a function of polarization state and phase can reveal the densities, relative brightness, lifetimes, and correlation times of different states. A two-population model was simulated, showing differing behavior qualitatively depending on model complexity. Application to an experimental sample mimicking domains or oligomerization revealed distinct coupling between brightness, lifetime, and correlation time.

## Figures and Tables

**Table 1:** Qualitative model-dependent features of N versus phase and N_perp_ versus phase plots for two populations of clusters. Population 1 clusters had a brightness of 3 and were dispersed at a cluster density of 0.39 clusters/beam area, while population 2 had a brightness of 1 and a cluster density of 0.40 clusters/beam area. Numerical values of the lifetimes and correlation times are given in the previous figures.

Model	Lifetime	Correlation Time	N/Nperp
1 τ and 1 ϕ	τ	ϕ	N is constant
			N_perp_ is constant
1 τ and 2 ϕ	τ_1_ = τ_2_	ϕ_1_, ϕ_2_	N is constant
			N_perp_ = F(ϕ_1_, ϕ_2_)
2 τ and 1 ϕ	τ_1_, τ_2_	ϕ	N has a peak N_perp_ broad, split
2 τ and 2 ϕ (non-assoc)	τ_1_, τ_2_	ϕ_1_, ϕ_2_	N has a peak N_perp_ broad, split
2 τ and 2 ϕ (assoc)	τ_1_, τ_2_	ϕ_1_, ϕ_2_	N has a peak N_perp_ broad, split, amplitude

**Table 2:** Models and goodness-of-fit parameters (AIC) describing the phase-sensitive fluorescence ICS data for the fluorescent beads (data in Figure 7).

Particles	Lifetime	Correlation Time	AIC
N = 0.65, B = 1	τ = 5.2 ns	None	−45
N_1_ = 0.49, N_2_ = 0.12, B_1_ = 1, B_2_ = 1	τ_1_ = 5.2, τ_2_ = 5.2 ns	None	−41
N_1_ = 0.38, N_2_ = 0.42, B_1_ = 3, B_2_ = 1	τ_1_ = 3.9 ns, τ_2_ = 19.4 ns	None	−113

**Table 3:** Models and goodness-of-fit parameters (AIC) describing the polarized phase-sensitive fluorescence ICS data for the fluorescent beads (data in Figure 7).

Particles	Lifetime	Correlation Time	AIC
N_1_ = 0.38, N_2_ = 0.42, B_1_ = 3, B_2_ = 1	τ_1_ = 3.9 ns, τ_2_ = 19.4 ns	ϕ_1_ = ϕ_2_ = 25.9 ns	−66
N_1_ = 0.33, N_2_ = 0.37, B_1_ = 3, B_2_ = 1	τ_1_ = 3.9 ns, τ_2_ = 19.4 ns	ϕ_1_ =1000 ns, ϕ_2_ = 0.01 ns	−90
N_1_ = 0.38, N_2_ = 0.42, B_1_ = 3, B_2_ = 1	τ_1_ = 3.9 ns, τ_2_ = 19.4 ns	(ϕ_1_, ϕ_2_, f_1_) and (ϕ_1_, ϕ_2_, f_2_)	−64

## Data Availability

The original contributions presented in this study are included in the article. Further inquiries can be directed to the corresponding author.

## Abbreviations

The following abbreviations are used in this manuscript:

FLIM	Fluorescence lifetime imaging microscopy
ICS	Image Correlation Spectroscopy
N	Cluster Density
B	Brightness

## Appendix A

In this appendix, formulae pertaining to FLIM and FLIM with polarized excitation/perpendicular detection are outlined. We begin with the simplest form of intensity decay, namely single exponential decay, characterized with lifetime, τ.

(A1) I(t) = I_o_ exp(−t/τ)

To represent the intensity decay in the frequency domain it is convenient to compute the sine and cosine transforms of the intensity decay given in Equation (A1).

(A2) Mcos(Φ) = ∫ I(t) cos(ωt) dt/∫ I(t) dt = 1/(1 + (ωτ)^2^

(A3) Msin(Φ) = ∫ I(t) sin(ωt) dt/∫ I(t) dt = ωτ/(1 + (ωτ)^2^

where ω is the modulation frequency.

If the intensity decay, I(t) is more complex, i.e., two components, with lifetimes τ_1_ and τ_2_ and associated amplitudes A_1_ and A_2_,

(A4) I(t) = I_o_ (A_1_ exp(−t/τ_1_) + A_2_ exp(−t/τ_2_)

Then the sine and cosine transforms become,

(A5) Mcos(ϕ) = ∫ I(t) cos(ωt) dt/∫ I(t) dt = α/(1 + (ωτ_1_)^2^) + (1 − α)/(1 + (ωτ_2_)^2^)

(A6) Msin(ϕ) = ∫ I(t) sin(ωt) dt/∫ I(t) dt = α ωτ_1_/(1 + (ωτ_1_)^2^) + (1 − α) ωτ_2_/(1 + (ωτ_2_)^2^)

In Equations (A5) and (A6) the fractions of the two states are expressed as fractional fluorescence, α = (A_1_τ_1_)/(A_1_τ_1_ + A_2_τ_2_).

Now we consider the situation of polarized excitation with perpendicular polarized emission. The intensity decay is now influenced by the decay of the excited state I(t) and the decay of anisotropy, r(t). The perpendicular-polarized component of the emission is given by I_perp_(t),

(A7) I_perp_(t) = (1/3) I(t) (1 − r(t))

If we assume the simplest form of anisotropy decay, r(t), characterized by a rotational correlation time ϕ and an initial, zero-time anisotropy of r_0_,

(A8) r(t) = r_0_ exp(−t/ϕ)

Then I_perp_(t) takes on the form of a double exponential decay in time,

(A9) I_perp_(t) = (1/3) I_o_ ((exp(−t/τ) − r_o_ exp(−t/τ_2_))

where τ_2_= τ/(1 + (τ/ϕ))

The cosine transform and sine transform of the perpendicular-polarized component of the emission are given by Equations (A5) and (A6), with τ_2_ = τ/(1 + (τ/ϕ)) and α = τ/(τ− r_o_ τ_2_); (1 − α) = −r_0_ τ_2_/(τ − r_o_ τ_2_).

To connect the cosine transform and sine transforms to the phase-sensitive fluorescence ICS and polarized phase-sensitive ICS, we make use of the trigonometric identity,

(A10) cos(x-a) = cos(x)cos(a) + sin(x)sin(a)

Substituting Equation (A10) into Equation (5) from the main text, we obtain

(A11) Bi(ϑ) = B_i_ (1 + mcos(Φ_i_ − ϑ)) = B_i_(1 + mcos(Φ)cos(ϑ) + msin(Φ)sin(ϑ))
